# Association between dietary vitamin E and osteoporosis in older adults in the United States

**DOI:** 10.3389/fendo.2024.1410581

**Published:** 2024-10-21

**Authors:** Ruoyu Zhuang, Wei Hou, Ting Zhang, Tao Wang

**Affiliations:** ^1^ Shanghai University of Traditional Chinese Medicine, Shanghai, China; ^2^ Department of Orthopedics and Traumatology, Longhua Hospital Shanghai University of Traditional Chinese Medicine, Shanghai, China

**Keywords:** aging, nutrition, osteoporosis, vitamin E, NHANES

## Abstract

**Background:**

Increased oxidative stress due to aging can lead to increased bone loss. The most abundant form of vitamin E, namely α-tocopherol, has high antioxidant properties and biological activity; however, its effect on osteoporosis has not been well studied in humans. We aimed to investigate the association between dietary vitamin E (α-tocopherol) and osteoporosis among older adults in the United States.

**Methods:**

This cross-sectional study analyzed data on older adults in the United States aged ≥50 years from the 2007–2010, 2013–2014 and 2017–2020 pre-pandemic cycles of the National Health and Nutrition Examination Survey. Sample-weighted multivariate regression models were used, with adjustments for relevant confounders.

**Results:**

This study comprised 5,800 individuals with available data on dietary intake and bone mineral density of hip and spine. The mean participant age was 61.4 (standard deviation, 8.7) years, and approximately 9.9% had osteoporosis. High vitamin E intake was significantly associated with a reduced risk of osteoporosis (odds ratio, 0.96, 95% confidence interval, 0.93–0.98). In addition, there was evidence of interaction between dietary vitamin E and prior fracture on preventing osteoporosis.

**Conclusions:**

Our study indicated a linear association between dietary vitamin E levels and osteoporosis in an older population in the United States. Further research is required to explore the potential effects of different forms of vitamin E on osteoporosis.

## 1 Introduction

Osteoporosis is defined as a systemic skeletal disease characterized by low bone mass and microarchitectural deterioration of bone tissue, with a consequent increase in bone fragility and susceptibility to fracture (1). According to World Health Organization (WHO) criteria, osteoporosis affects approximately 6% of men and 21% of women aged ≥50 years (2), resulting in >9 million osteoporotic fractures annually (3). Previous studies have indicated that oxidative stress with aging is the main protagonist in the fundamental mechanism of bone loss (4, 5); thus, diets rich in antioxidants are reported to contribute to the prevention of osteoporosis (6).

Vitamin E is a lipophilic nutrient that naturally occurs in eight isoforms, including α, β, γ, and δ isomers of tocopherols (TFs) and tocotrienols (T3s), mainly found in plant oils, seeds, nuts, cereals, fruit, and vegetables (7). Both TFs and T3s are well known for their potent antioxidant properties, capable of scavenging reactive oxygen species (ROS) and free radicals (8). Within the TF group, α- TF present the highest antioxidant activity followed by other isomers (9). Despite the similar structure and antioxidant activity, vitamin E isoforms differ greatly in bioavailability and metabolism (10). While all isoforms are biologically active, only α-tocopherol is preferentially recognized by the α-tocopherol transfer protein (α-TTP) and retained at high levels in plasma and tissues (11, 12). α-TF has been reported to play a role in protecting against bone loss owing to oxidative stress, which is induced by estrogen deficiency or free radicals (13, 14).

However, the effects of vitamin E on the risk of osteoporosis remain unclear. Animal studies have shown that vitamin E (α-TF) in high doses could prevent osteoporosis in stressful conditions but might exert harmful effects in normal conditions (15, 16). In human studies, adverse effects have been rarely observed, while a positive relationship between vitamin E and bone mineral density (BMD) has been reported (17, 18).

While several studies have evaluated the association between α-TF with BMD, or bone turnover markers (19, 20), few studies have explored the association between TF and osteoporosis. In this cross-sectional study, we aimed to investigate the association between dietary vitamin E (α-TF) and osteoporosis using data derived from the United States National Health and Nutrition Examination Survey (NHANES).

## 2 Methods

### 2.1 Data source

The NHANES is a nationally representative program of the National Center for Health Statistics (NCHS) designed to assess the health and nutritional status of the civilian, non-institutionalized United States population using a complex, multistage probability sampling design (21). BMD data concerning the femur and spine were only available in the NHANES 2005–2010 cycles for individuals aged ≥8 years, 2013–2014 cycle for adults aged ≥40 years and in the 2017–2020 pre-pandemic cycle for adults aged ≥50 years. Information in relation to the intake of vitamin D and dietary supplements has only been available since the 2007–2008 cycle. Therefore, de-identified data for individuals aged ≥50 years were extracted from the 2007–2010, 2013–2014 and 2017–2020 pre-pandemic cycles. The NCHS Ethics Review Board approved the NHANES protocols, and written informed consent was obtained from all participants. This study adhered to the Strengthening the Reporting of Observational Studies in Epidemiology guidelines.

### 2.2 Study design and population

Data for our cross-sectional study were gathered from participants during the NHANES cycles over 9 years (2007–2010, 2013–2014 and 2017–2020 pre-pandemic). The exclusion criteria were as follows: age <50 years; unavailable data on BMD of the total hip, femoral neck, and lumbar spine; unavailable data on vitamin E; and unavailable data on covariates, including age, sex, race/ethnicity, education level, dietary supplements (vitamin D and calcium), body mass index (BMI), smoking status, prior fracture, hormone use (prednisone or cortisone), and physical activity.

### 2.3 Definition of dietary vitamin E (α-TF)

Nutrient intake information was collected through a dietary recall interview at a mobile examination center regarding the types and amounts of foods and beverages consumed within the previous 24-h period. The dietary interview component was conducted as a partnership between the United States Department of Agriculture (USDA) and the United States Department of Health and Human Services (DHHS). The intake of vitamin E from foods and beverages was calculated using USDA’s Food and Nutrient Databases for Dietary Studies (FNDDS) for the corresponding NHANES (22). Currently, most nutrient databases do not distinguish between different isoforms of vitamin E. In the dietary data files, vitamin E was recorded only in the form of α-TF. Data on other equivalents and vitamin E supplements were unavailable in the NHANES database. In the United States, the Recommended Dietary Allowance (RDA) for vitamin E is 15 mg/day of α-TF in adults for both men and women (23).

### 2.4 Diagnosis of osteoporosis

BMD data of the hip and spine was obtained using dual-energy X-ray absorptiometry (DXA) scans on Hologic densitometers (Hologic, Inc., Bedford, Massachusetts). Currently, DXA is the most widely accepted approach for measuring BMD, given its ease of use and low radiation doses (24, 25). For adults aged ≥50 years, osteoporosis is generally classified by a BMD value that is ≥2.5 standard deviations (SDs) below the mean value of a young female adult, at the total femur, femoral neck, or lumbar spine (26, 27). The mean BMD and SD of the total hip and femoral neck were based on data from non-Hispanic white women aged 20–29 years in the Third NHANES (NHANES III) database (28). Similarly, the mean BMD and SD of lumbar spine were based on data from the Hologic reference database (29).

### 2.5 Covariates

Based on the published literature and clinical experience, the following covariates were included: age, sex, race/ethnicity, education level, BMI, smoking status, prior fracture, hormone use, physical activity, energy intake, vitamin D intake, calcium intake, vitamin D supplementation and calcium supplementation (30, 31). Race/ethnicity was divided into five categories, namely Mexican American, non-Hispanic white, non-Hispanic black, other Hispanic, and other race (including multiracial). Education level was divided into four categories: lower than high school, high school or equivalent, some college or equivalent, college graduate or higher. As calculated using the NHANES, BMI was obtained from body measurement data. Smoking status was divided into three groups: never smoked (or smoked <100 cigarettes), former smoker (smoked ≥100 cigarettes but had quit smoking), and current smoker. Prior fracture was determined using the survey question: “Has a doctor ever informed you that you had broken or fractured your hip/wrist/spine?” Hormone use was based on another survey question: “Have you ever taken any prednisone or cortisone medication nearly every day for ≥1 month?” Physical activity was evaluated using weekly metabolic equivalent task (MET)-minute aggregated scores to quantify energy expenditure (32). The NHANES classifications were used to calculate MET-minute scores based on the following formula: suggested MET scores × minutes of corresponding activity (33). Data concerning the intake of energy, vitamin D, and calcium were collected with vitamin E through the dietary recall interview. The intake of vitamin D and calcium supplements depended on the dietary records of supplement use during the 30 days prior to the survey date.

### 2.6 Statistical analysis

According to NHANES analytic guidelines, dietary sample weights across the survey cycles totaling 9.2 years were selected and constructed appropriately based on data from the 24-h dietary recall to account for the complex sample design and non-response (34). The participants’ characteristics were presented as weighted mean (SD) for continuous variables and as unweighted frequency (weighted percentage) for categorical variables. The differences between groups were tested using one-way analyses of variance (ANOVA) for continuous variables and chi-squared tests for categorical variables.

Odds ratios (ORs) and 95% confidence intervals (CIs) were calculated to assess the association between α-TF and osteoporosis using logistic regression models. Model 1 was adjusted for sociodemographic variables (sex, age, race/ethnicity, and education level). Model 2 was based on model 1 and the risk factors for osteoporosis (BMI, smoking status, prior fracture, hormone use, and physical activity). Model 3 was adjusted from model 2 to include dietary factors for osteoporosis (energy intake, vitamin D intake, calcium intake, vitamin D supplementation and calcium supplementation). Restricted cubic spline (RCS) was performed with four knots at the 5th, 35th, 65th, and 95th percentiles of dietary vitamin E consumption to assess linearity. The dose-response curve between dietary vitamin E levels and osteoporosis was plotted based on the covariates in model 3.

In addition, subgroup analyses were performed according to sex, age, race/ethnicity, education level, BMI, smoking status, prior fracture, hormone use, vitamin D supplementation and calcium supplementation, and interactions were tested with likelihood ratio test. Sensitivity analyses were performed to assess the robustness of our findings. First, considering the potential effects of other important nutrients, the models were additionally adjusted for factors beneficial to bone health, such as protein (35), vitamin K (36, 37), magnesium (38), and zinc (39), as well as detrimental factors, such as caffeine (40) and alcohol (41). Second, due to vitamin E absorption being affected by other antioxidants and fat, vitamin C and dietary polyunsaturated fat (PUF) (42) were also included in the models. Third, given that food intake often varies according to the type and amount between weekdays and weekends, we conducted sensitivity analyses using intake data from the second dietary recall interview, which was collected by telephone 3–10 days later.

All statistical analyses were performed with R, version 4.3.2 (R Project for Statistical Computing) software using the survey package, version 4.2.1, and with Free Software Foundation Statistics software, version 1.9.2. In all tests, *P <*0.05 (two-sided) was considered to indicate statistical significance.

## 3 Results

### 3.1 Participants’ characteristics

**Figure 1:**
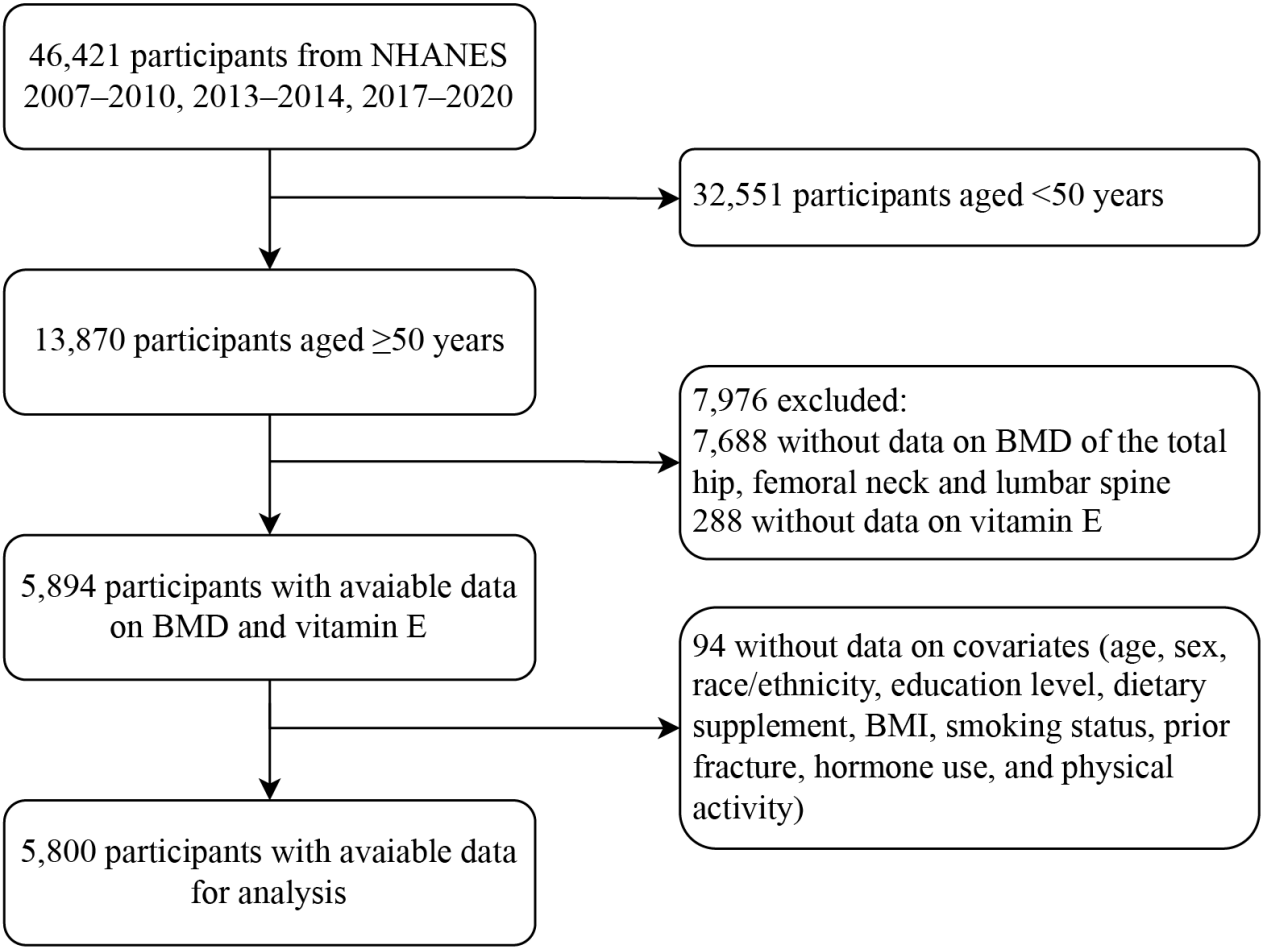
Flow diagram of the screening and enrollment of study participants. NHANES, National Health and Nutrition Examination Survey; BMD, bone mineral density; BMI, body mass index.

Among 46,421 participants from the multiyear survey cycles, a total of 13,870 adults aged ≥50 years were included, and 5,894 had valid data concerning DXA examinations and dietary interviews. In total, 5,800 individuals with available data on covariates remained eligible for the analysis (**Figure 1**). Their characteristics are presented according to quartiles of dietary vitamin E levels (**Table 1**). Based on the weighted analyses, the mean age was 61.4 (SD, 8.7) years, and 3,000 (54.8%) were female. Based on BMD data and WHO standards for osteoporosis, 634 (9.9%) participants were diagnosed with osteoporosis. The prevalence of osteoporosis was lower among the participants with the highest (quartile 4) vitamin E intake than those with the lowest (quartile 1) intake. Participants with a higher intake of vitamin E were more likely to be male, non-Hispanic white, and have a college education or higher. Moreover, a higher intake of vitamin E was associated with higher consumption of vitamin D, calcium, and their supplements.

**Table 1:** Characteristics of study participants by dietary vitamin E.

Characteristics	Dietary Vitamin E (mg/day)					*P* value
	Total	Quartile 1 (≤4.08)	Quartile 2 (4.09–6.48)	Quartile 3 (6.49–9.84)	Quartile 4 (≥9.85)	
No.	5800	1447	1449	1450	1454	
Gender, n (%)						<0.001
Male	2800 (45.2)	626 (40.3)	629 (38.7)	735 (46.6)	810 (52.3)	
Female	3000 (54.8)	821 (59.7)	820 (61.3)	715 (53.4)	644 (47.7)	
Age (year), Mean (SD)	61.4 (8.7)	62.2 (9.1)	62.2 (8.5)	61.7 (8.8)	60.1 (8.2)	<0.001
Race/ethnicity, n (%)						<0.001
Mexican American	833 (5.7)	251 (8.1)	208 (5.2)	201 (5.4)	173 (4.8)	
Other Hispanic	649 (5.0)	198 (7.4)	181 (5.4)	162 (5.0)	108 (3.1)	
Non-Hispanic white	2529 (71.9)	544 (64.5)	620 (70.7)	636 (72.0)	729 (77.4)	
Non-Hispanic black	1234 (10.0)	338 (12.9)	297 (9.8)	299 (10.0)	300 (8.4)	
Other race	555 (7.5)	116 (7.2)	143 (9.0)	152 (7.7)	144 (6.3)	
Education level, n (%)						<0.001
Lower than high school	1504 (14.9)	565 (26.0)	369 (14.3)	328 (11.8)	242 (10.7)	
High school or equivalent	1354 (25.5)	349 (27.4)	371 (28.5)	321 (24.1)	313 (23.2)	
Some college or equivalent	1595 (28.7)	324 (25.4)	401 (29.7)	426 (31.1)	444 (28.0)	
College graduate or higher	1347 (30.9)	209 (21.2)	308 (27.5)	375 (33.0)	455 (38.1)	
BMI (kg/m^2^), Mean (SD)	28.4 (5.6)	28.6 (5.8)	28.4 (5.7)	28.5 (5.7)	28.1 (5.4)	0.543
Smoking status, n (%)						<0.001
Never	3080 (54.0)	749 (50.1)	788 (53.5)	779 (56.4)	764 (54.8)	
Former	1777 (31.0)	383 (27.7)	456 (32.1)	459 (29.9)	479 (33.3)	
Current	943 (15.0)	315 (22.2)	205 (14.3)	212 (13.7)	211 (11.9)	
Prior fracture, n (%)	704 (15.1)	173 (15.2)	172 (15.1)	177 (14.1)	182 (15.6)	0.919
Hormone use, n (%)	359 (6.4)	99 (8.1)	88 (6.5)	92 (6.7)	80 (4.8)	0.056
MET-minute scores, Median (IQR)	300.0 (40.0, 960.0)	200.0 (0.0, 739.2)	240.0 (0.0, 900.0)	360.0 (60.0, 960.0)	420.0 (97.7, 1,000.0)	<0.001
Total femur BMD (mg/cm^2^), Mean (SD)	918.4 (157.2)	902.0 (161.1)	909.0 (153.5)	922.0 (151.6)	933.3 (160.5)	0.032
Femoral neck BMD (mg/cm^2^), Mean (SD)	762.2 (137.3)	751.8 (140.2)	756.2 (135.4)	762.7 (134.5)	773.1 (138.6)	0.139
Lumbar spine BMD (mg/cm^2^), Mean (SD)	999.9 (163.1)	978.0 (166.1)	998.1 (165.0)	1004.9 (156.4)	1011.3 (163.6)	0.006
Osteoporosis, n (%)	634 (9.9)	206 (13.6)	169 (10.6)	131 (8.8)	128 (7.8)	<0.001
Energy intake (kcal/day), Mean (SD)	2004.3 (860.5)	1299.8 (501.1)	1748.9 (547.9)	2094.8 (661.1)	2580.2 (967.2)	<0.001
Vitamin D intake (μg/day), Median (IQR)	3.2 (1.5, 5.8)	2.1 (0.8, 4.2)	3.0 (1.4, 5.2)	3.2 (1.7, 5.8)	4.1 (2.1, 7.6)	<0.001
Calcium intake (mg/day), Mean (SD)	909.2 (532.3)	625.6 (398.0)	817.1 (428.5)	923.3 (456.1)	1150.7 (621.1)	<0.001
Vitamin D supplementation, n (%)	2562 (50.7)	506 (41.0)	636 (50.0)	666 (51.3)	754 (57.0)	<0.001
Calcium supplementation, n (%)	2434 (48.1)	495 (39.9)	591 (47.5)	626 (48.7)	722 (53.3)	<0.001

BMD, bone mineral density; BMI, body mass index; MET, metabolic equivalent task; SD, standard deviation; IQR, interquartile range.

Data are presented as weighted mean (SD) or median (IQR) for continuous variables, and as unweighted number (weighted percentage) for categorical variables.

### 3.2 Multivariate regression analyses

The results of the sample-weighted multivariate regression analyses are presented in **Table 2**. As a continuous variable, dietary vitamin E (α-TF) was significantly associated with osteoporosis (OR, 0.95, 95% CI, 0.93–0.97) in the crude model, and the association remained significant among the adjusted models. A higher intake of vitamin E was associated with a lower risk of osteoporosis (OR, 0.61, 95% CI, 0.41–0.92) in the comparison between quartile 4 and quartile 1 after full adjustment (model 3), showing a linear relationship. Each additional 1 mg of daily vitamin E intake was associated with a 4% lower osteoporosis risk (OR, 0.96, 95% CI, 0.93–0.98). Dietary vitamin E levels and the risk of osteoporosis had a negative linear relationship as shown in RCS (**Supplementary Figure 1**).

**Table 2:** Association between dietary Vitamin E and osteoporosis.

Variable	OR (95% CI)					*P* value
	Quartile 1 (≤4.08)	Quartile 2 (4.09–6.48)	Quartile 3 (6.49–9.84)	Quartile 4 (≥9.85)	Vitamin E (mg/day)	
Crude	1 (Ref)	0.75 (0.56–1.00)	0.62 (0.44–0.86)	0.54 (0.39–0.74)	0.95 (0.93–0.97)	<0.001
Model 1	1 (Ref)	0.75 (0.55–1.03)	0.70 (0.48–1.02)	0.73 (0.52–1.03)	0.97 (0.95–1.00)	0.026
Model 2	1 (Ref)	0.75 (0.52–1.07)	0.70 (0.46–1.06)	0.71 (0.51–0.98)	0.97 (0.95–0.99)	0.004
Model 3	1 (Ref)	0.70 (0.48–1.03)	0.64 (0.40–1.03)	0.61 (0.41–0.92)	0.96 (0.93–0.98)	0.002

OR, odds ratio; CI, confidence interval; Ref, reference; BMI, body mass index; MET, metabolic equivalent task.

Model 1 was adjusted for sex, age, race/ethnicity, education level.

Model 2 was adjusted for model 1, BMI, smoking status, prior fracture, hormone use, MET-minute scores.

Model 3 was adjusted for model 2, energy intake, vitamin D intake, calcium intake, vitamin D supplementation, and calcium supplementation.

### 3.3 Subgroup analyses

**Figure 2:**
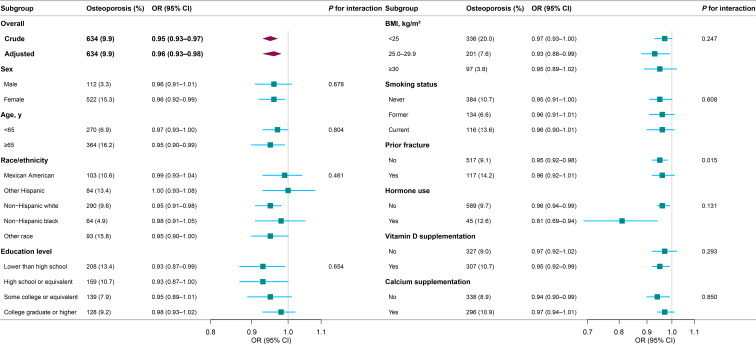
Subgroup analyses for the association between dietary vitamin E and osteoporosis. Each stratification was adjusted for sex, age, race/ethnicity, education level, BMI, smoking status, prior fracture, hormone use, MET-minute scores, energy intake, vitamin D intake, calcium intake, vitamin D supplementation, and calcium supplementation, except the stratification factor itself. Osteoporosis events are presented as unweighted number (weighted percentage). OR, odds ratio; CI, confidence interval; BMI, body mass index; MET, metabolic equivalent task.

The results of the subgroup analyses are presented in **Figure 2**. The association between dietary vitamin E and osteoporosis was consistent across subgroups according to sex, age, race/ethnicity, education level, BMI, smoking status, prior fracture, hormone use, vitamin D supplementation and calcium supplementation. In participants with a history of hormone use, vitamin E intake was associated with lower osteoporosis risk (OR, 0.81, 95% CI, 0.69–0.94). In the fully adjusted model, an interaction was observed between vitamin E and prior fracture.

### 3.4 Sensitivity analyses

Following further adjustment for other nutrients affecting bone health and factors related to vitamin E absorption (**Supplementary Table 1**), the results of the sensitivity analyses for vitamin E were similar to those of the primary findings. The linear association between dietary vitamin E and osteoporosis was reinforced to a degree in the fully adjusted model (OR, 0.91, 95% CI, 0.87–0.95) when both days of dietary intake were combined (**Supplementary Table 2**).

## 4 Discussion

In this nationally representative cross-sectional study involving 5,800 older adults in the United States, we found that a higher vitamin E intake, measured using a 24-h dietary recall interview, was significantly associated with a lower osteoporosis risk in a dose-response manner. In our subgroup analyses, the OR for the association between dietary vitamin E and osteoporosis was lower among participants with a history of hormone use, and there was an interaction between dietary vitamin E and prior fracture. After adjusting for various nutrients related to bone health, and the aggregation of intake data from the 2-day interview, the linear association between vitamin E and osteoporosis remained essentially unchanged in the sensitivity analyses.

Several human studies have reported a positive relationship between vitamin E intake and bone health, based on BMD. Two cross-sectional studies reported that greater consumption of vitamin E was associated with greater BMD, mainly in the lumbar spine, in middle-aged Asian women (43, 44). These results accord with our findings; however, the participants in those studies were recruited only from the local community or from clinics, which may have limited their external validity. Our study used NHANES data to obtain nationally generalizable estimates. Moreover, while most studies have considered the association between dietary vitamin E and BMD, few have examined osteoporosis, which is a clear indicator of bone loss and fracture risk. This study is the first to report a linear relationship between dietary vitamin E levels and osteoporosis in a general population of older American participants.

To our knowledge, two previous studies have investigated the association between dietary vitamin E and BMD, with conflicting results. A longitudinal study indicated that a greater loss of femoral neck BMD was associated with increased intake of vitamin E, as a surrogate marker for polyunsaturated fatty acids (45), whereas another cross-sectional study found no significant association between vitamin E intake and BMD at any site among the women in the United States, where most participants had a healthy BMD (46). Similarly, a recent study has reported no significant association between vitamin E intake and osteoporosis, with a lower prevalence of osteoporosis (47). These varying results are likely due to the effects of confounding variables and sampling errors. To address such limitations, we combined five survey cycles from the NHANES database involving an older population with available BMD data. According to WHO criteria, the weighted osteoporosis prevalence was 9.9% in our study, which was comparable to the trends in osteoporosis prevalence from 2007–2008 to 2017–2018 (9.4%–12.6%) in NCHS report (48). Confounding factors for vitamin E absorption were adjusted in the sensitivity analyses.

No previous studies have investigated the association between vitamin E levels and osteoporosis in different subgroups. In our subgroup analyses, there was a significant interaction between dietary vitamin E and osteoporosis among individuals with or without previous fractures. Regardless of prior fractures, vitamin E represented a protective factor partly due to its improvement in post-fracture healing (49) and physical function (50). However, considering the limited sample size in our study and the potential influence of confounding factors, it is essential to interpret these findings with caution. Additional prospective studies are warranted for further exploration.

Animal studies have shown that vitamin E plays an important role in regulating bone metabolism and preventing bone loss. Vitamin E, in the forms of both α-TF and T3, has been found to improve both static and dynamic bone histomorphometry parameters, while α-TF has been found to exerted similar or inferior effects to T3 in preserving bone microarchitecture (51, 52). One study reported that ex vivo osteoclast numbers in ovariectomized rats were suppressed with three doses of α-TF, and most effectively with the lowest dose (53). Other studies have reported that no bone loss was observed with high dietary α-TF in different models of rats and mice (54–56). These results accord with our findings.

This study had a few limitations. Owing to its cross-sectional design, the causal relationship between vitamin E intake and osteoporosis could not be determined. Therefore, well-designed cohort studies are warranted. In this observational study, we were unable to rule out potential residual confounding factors, such as other dietary factors, other common disorders affecting the bone, and serum biomarkers, for which data were available in only some survey cycles. With incomplete information on times and reasons of fractures, prior fractures could not be defined entirely as osteoporotic. The association between different vitamin E homologs and osteoporosis remains unclear. Given that our study was based on a United States aged ≥50 years, caution is needed when generalizing to other populations and other age groups.

## 5 Conclusion

Our study showed a significant linear association between dietary vitamin E levels and osteoporosis in an older population in the United States. Further research is required to explore the potential effects of different forms of vitamin E on osteoporosis.

## Data Availability

Publicly available datasets were analyzed in this study. This data can be found here: https://www.cdc.gov/nchs/nhanes/index.htm.
