# Supplementary material for: Association between dietary vitamin E and osteoporosis in older adults in the United States
Source: Front Endocrinol (Lausanne). 2024 Oct 21;15:1410581. doi: 10.3389/fendo.2024.1410581 (PMC11532079; doi:10.3389/fendo.2024.1410581)
Supplement: Supplementary file 1 [file DataSheet1.docx]

**Supplementary Materials**

**Supplementary Table 1** Sensitivity analyses for the association between dietary vitamin E and osteoporosis (adjusted for other nutrients).

| Variable | OR (95% CI) | | | | | *P* value |
| --- | --- | --- | --- | --- | --- | --- |
|  | Quartile 1 | Quartile 2 | Quartile 3 | Quartile 4 | Vitamin E (mg/day) |  |
| Primary analysis | 1 (Ref) | 0.70 (0.48–1.03) | 0.64 (0.40–1.03) | 0.61 (0.41–0.92) | 0.96 (0.93–0.98) | 0.002 |
| Additional adjustment: | | | | | | |
| Protein, vitamin K, magnesium and zinc | 1 (Ref) | 0.72 (0.49–1.06) | 0.68 (0.42–1.11) | 0.69 (0.47–1.03) | 0.96 (0.94–0.99) | 0.006 |
| Caffeine and alcohol | 1 (Ref) | 0.69 (0.47–1.02) | 0.62 (0.38–1.00) | 0.57 (0.38–0.87) | 0.95 (0.93–0.98) | 0.002 |
| Vitamin C and polyunsaturated fat | 1 (Ref) | 0.73 (0.50–1.08) | 0.70 (0.41–1.19) | 0.74 (0.43–1.26) | 0.96 (0.93–1.00) | 0.027 |

Abbreviations: OR, odds ratio; CI, confidence interval; Ref, reference.

Covariates adjusted in the models were consistent with the fully adjusted models for total unless otherwise specified.

**Supplementary Table 2** Sensitivity analyses for the association between dietary vitamin E and osteoporosis (including the second dietary recall).

| Variable | OR (95% CI) | | | | | *P* value |
| --- | --- | --- | --- | --- | --- | --- |
|  | Quartile 1  (≤4.55) | Quartile 2  (4.56–6.67) | Quartile 3  (6.68–9.62) | Quartile 4  (≥9.63) | Vitamin E (mg/day) |  |
| Crude | 1 (Ref) | 0.63 (0.41–0.96) | 0.59 (0.37–0.94) | 0.41 (0.28–0.59) | 0.92 (0.89–0.95) | <0.001 |
| Model 1 | 1 (Ref) | 0.73 (0.45–1.16) | 0.75 (0.45–1.24) | 0.58 (0.39–0.86) | 0.95 (0.92–0.98) | 0.003 |
| Model 2 | 1 (Ref) | 0.74 (0.45–1.20) | 0.73 (0.45–1.19) | 0.52 (0.34–0.80) | 0.94 (0.91–0.97) | <0.001 |
| Model 3 | 1 (Ref) | 0.68 (0.40–1.15) | 0.61 (0.35–1.06) | 0.39 (0.22–0.69) | 0.91 (0.87–0.95) | <0.001 |

Abbreviations: OR, odds ratio; CI, confidence interval; Ref, reference; BMI, body mass index; MET, metabolic equivalent task.

Model 1 was adjusted for sex, age, race/ethnicity, education level.

Model 2 was adjusted for model 1, BMI, smoking status, prior fracture, hormone use, MET-minute scores.

Model 3 was adjusted for model 2, energy intake, vitamin D intake, calcium intake, vitamin D supplementation, and calcium supplementation.


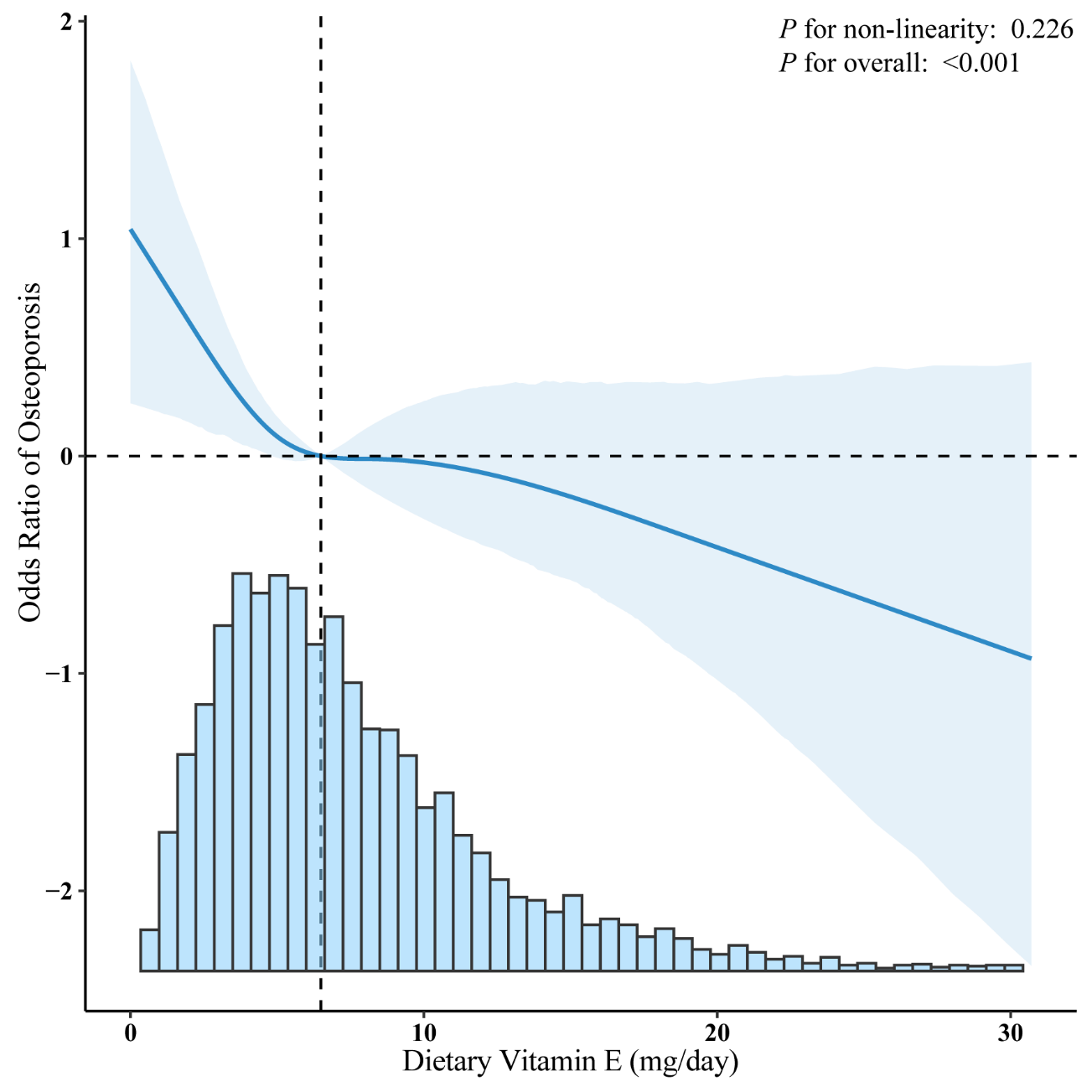


**Supplementary Figure 1** Restricted cubic spline of the association between dietary vitamin E and osteoporosis. The solid and dashed lines represent the OR and 95% CI. Only 99% of the data is displayed, adjusted for sex, age, race/ethnicity, education level, BMI, smoking status, prior fracture, hormone use, MET-minute scores, energy intake, vitamin D intake, calcium intake, vitamin D supplementation, and calcium supplementation. Abbreviations: OR, odds ratio; CI, confidence interval; BMI, body mass index; MET, metabolic equivalent task.
